# Viewpoint-Driven Simplification of Plant and Tree Foliage

**DOI:** 10.3390/e20040213

**Published:** 2018-03-21

**Authors:** Cristina Gasch, Inmaculada Remolar, Miguel Chover, Cristina Rebollo

**Affiliations:** Institute of New Image Technologies, Universitat Jaume I, Castellón E-12071, Spain

**Keywords:** geometric simplification, vegetation visualization, mutual information

## Abstract

Plants and trees are an essential part of outdoor scenes. They are represented by such a vast number of polygons that performing real-time visualization is still a problem in spite of the advantages of the hardware. Some methods have appeared to solve this drawback based on point- or image-based rendering. However, geometry representation is required in some interactive applications. This work presents a simplification method that deals with the geometry of the foliage, reducing the number of primitives that represent these objects and making their interactive visualization possible. It is based on an image-based simplification that establishes an order of leaf pruning and reduces the complexity of the canopies of trees and plants. The proposed simplification method is viewpoint-driven and uses the mutual information in order to choose the leaf to prune. Moreover, this simplification method avoids the pruned appearance of the tree that is usually produced when a foliage representation is formed by a reduced number of leaves. The error introduced every time a leaf is pruned is compensated for if the size of the nearest leaf is altered to preserve the leafy appearance of the foliage. Results demonstrate the good quality and time performance of the presented work.

## 1. Introduction

Outdoor scenes are very common in many interactive applications, such as video games or virtual reality walk-throughs. Vegetation is an essential part of these environments, producing a lack of realism if some plants do not appear in the scenes. Tree and plant modeling has been widely researched, and different techniques have appeared in the literature to represent these objects. However, real-time visualization of forest or environments with vegetation is still a problem in spite of the current development of the graphics hardware. The main reason for this is that tree models are formed by such a vast number of polygons that interactive visualization of scenes with vegetation is practically impossible.

The methods that have appeared to perform their real-time visualization can be mainly classified into two groups: image-based and geometry-based rendering. The methods in the first group, image-based rendering, change the geometry of the plant by an image that represents it. The advantage of these methods is that visualization time is drastically reduced compared to geometry rendering. However, the lack of realism is remarkable when the camera approaches the object. The other group in the classification, the geometry-based rendering, can be divided in turn into two groups: point-based rendering and rendering based on polygons. The first group represents the leaves by points or even lines when they are situated far from the viewer. The polygon-based rendering allows a realistic visualization even when the camera is really close to the plant. Some applications perform this rendering method because the detail of the leaves in the foliage is required. Its main drawback is the vast amount of polygons, which delays the interactive visualization if an optimization technique is not applied.

Multiresolution modeling [1,2] has been successfully presented as a solution to the problem of efficient manipulation of highly detailed polygonal surfaces. Discrete multiresolution modeling consists of representing an object by means of multiple approximations or levels of detail (LoDs) where each approximation or LoD represents the original object using a different number of polygons. Interactive applications manage this set of LoDs and change the approximation to be rendered in real time taking into account some parameters, such as the distance to the viewer or the amount of pixels the object occupies in the final image. Game engines, such as Unity 3D [3], allow game designers to use this solution by including a component called LOD Groupwhere different approximations of the same object can be uploaded (LOD1, LOD2, etc.), but only one of them will be rendered in the interactive application, depending on the established conditions. The distance to the camera is the most used criterion to change the rendered LoD.

Simplification methods deal with the task of reducing the amount of geometry in the meshes, without a drastic loss of appearance [4]. They allow us to generate the different LoDs that are necessary to create the set of approximations that compound a discrete multiresolution model [1,2]. Generally, these simplification techniques have been addressed to work with general continuous meshes. The trunks meet this type of representation. However, the green part of the plants and the foliage of the trees are not represented by these kinds of meshes. They are formed by independent polygons where the image of the leaf is textured on them. It has been demonstrated that general simplification methods do not properly work with isolated polygons [5]. These methods generally eliminate polygons, so that the appearance of the crown after an automatic process of simplification is that it has been pruned. The number of leaves is less than before, so the tree appears less leafy.

Some simplification methods specially addressed to this part of the plants have appeared in the literature, such as the one presented by Remolar et al. [6]. The authors propose the leaf collapse as a simplification operation: two leaves are transformed into a single one, so that the area of the new leaf is similar to the area initially formed by the two leaves. Other works, such as presented by Zhang and Blaise [7], deal with the same collapse operation; changing the criteria, they select the leaves to be collapsed by some measurements of similarity, such as the distance and diameter of the leaf.

Performing stochastic pruning is another way to obtain simplified approximations of the foliage, such as the works presented by Cook et al. [8,9]. In their work, some elements are pruned (i.e., eliminated), and the remaining elements are altered to preserve the overall appearance of the scene. The elements that are pruned are randomly chosen.

This paper presents a simplification method addressed to the foliage of the plants that is driven by the visual appearance. A viewpoint-based simplification method that manages an information-theoretic measure called viewpoint mutual information (VMI) [10] has been adapted to the geometric simplification of the sparse part of the plants. In our work, a set of cameras have been uniformly distributed around the foliage, and the simplification of every leaf is evaluated, checking the introduced error in every one of the 20 images obtained from the different cameras. Removing the leaves that introduce the lowest error makes it possible to increase the degree of simplification in hidden interiors because they do not have visual impact. This allows us to obtain a better preservation of the visible parts of the model. Another important feature of our algorithm is that the size of some remaining leaves is altered to preserve the leafy appearance of the representation of the tree canopy. Experimental results show that our method yields better visual performance, comparing the results with those produced by applying the method presented in [6].

This article is organized as follows. In Section 2, we survey the previous work of geometric simplification that deals with plants and trees, stressing the methods that use basic information-theoretic measures. Section 3 presents the metric used to choose the leaf that introduces the lowest error. Then, Section 4 analyses the presented simplification scheme. The results are shown and evaluated in Section 5, and finally, Section 6 exposes the conclusions of the work and some proposed future lines of research.

## 2. Related Work

This section reviews the methods that have appeared to reduce the complexity of the sparse component of the trees and plants. Although some representative works are based on points [5,11,12] or based on images [13,14,15], we will review those that are based on polygons, which is the primitive we use in our simplification method. Simplification methods that use the mutual information as a criterion to reduce the polygonal complexity are also reviewed in this section.

### 2.1. Simplification Methods of Tree Models

Among the most important works that can diminish the number of leaves in a crown while maintaining its appearance are those that make an iterative leaf collapse process stand out.

In 2002, Remolar et al. [6] proposed the first method for foliage simplification that deals with polygons, the foliage simplification algorithm (FSA). Two leaves disappear and are replaced by a new one that preserves an area similar to the original ones, maintaining the visual effect of the foliage. The method uses an error function to determine which pair of leaves is collapsed, taking the Hausdorff distance between two leaves and their planarity into account. One year later, Zhang et al. [7] presented a new algorithm, progressive leaves union (PLU), which enhances the cost of FSA by refining the error function with additional criteria. They also take the Hausdorff distance into account, but considering that the choice of the pair of leaves to collapse is reduced to leaves that have been collapsed with less than two different leaves (close in position and similar in shape). Neither FSA nor PLU can deal with quadrilaterally-shaped leaves. Based on PLU, Zhang et al. [16] propose a novel method, hierarchical union of organs (HUO). The method includes triangular leaves and introduces a hierarchy into simplification by making use of the fact that the leaves in a crown are constructed by instantiating a phyllotaxy cluster sample with different transformations. In 2009, Deng et al. [17] presented two different algorithms based on HUO, one for broad leaves represented by quadrilaterals and one for thin leaves represented by lines. They introduce leaf density to adapt compression to the local distribution of leaves, so that more visually-relevant details are kept. Two years later, Bao et al. [18] proposed a new leaf modeling method that uses the texture to simplify triangular mesh models of leaves.

Other works that have also appeared try to reduce the foliage by performing pruning operations. Based on stochastic pruning, Cook et al. [8,9] proposed a stochastic simplification of leaves with random removal of leaves. The geometry is reduced down to a certain fraction. Next, the remaining geometry is scaled so that the total area of rendered surfaces is equal to the original area. In 2011, Neubert et al. [19] presented an optimized pruning algorithm, improving upon previous techniques by applying model-specific geometry reduction and optimized scaling, as well as view-optimized pruning. They introduced precision and recall (PR) as a measure of quality for rendering complex geometry with pruning. This measure does not consider pixel colors, but the right pixels of a rendered object are set.

View-dependent representation techniques take into account the distance between tree models and viewpoints. These methods diminish the number of leaves in a crown in a non-uniform way, eliminating more leaves in the less visible parts of the tree than in the more visible areas.

In 2011, Gumbau et al. [20] presented a foliage pruning method for real-time vegetation rendering based on stochastic pruning of needless leaves for a given level of detail (LoD). In order to build the LoD scheme, a stochastic pruning method is applied in a pre-process; LoD tree model foliage is divided into a cloud of cells; and each cell’s visibility is computed from a set of external viewpoints surrounding the foliage. Following a camera-dependent criterion, the less visible parts of the foliage are detected in real time. The appropriate resolution of the foliage is calculated taking into account both the distance of the tree to the observer, as well as the visibility of the leaves.

Lindstrom and Turk [21] address the problem of visual similarity by developing a purely image-based metric. They determine the cost of an edge collapse operation by rendering the model from several viewpoints. The algorithm compares the rendered images to the original ones and adds the mean-square error in luminance across all the pixels of all the images. Then, all edges are sorted by the total error incurred in the images, and after that, the edge collapse that produces the least error is chosen. Its main disadvantage is the high temporal cost. In 2002, Zhang and Turk [22] proposed an algorithm that defines a visibility function between the surfaces of a model and a surrounding sphere of up to 258 cameras. A table of visibility between the camera positions and surface triangles is computed, obtaining for each triangle a measure of its visibility. In order to guide the simplification process, this visibility measure is combined with the quadric error measure introduced by Garland and Heckbert [23]. The visibility map calculation is a very time-consuming process, so this method has a high cost.

The simplification techniques developed by Lee et al. [24] and Lee and Kuo [25] are based on pixel-based metrics. They do not consider the tree model leaf density. To solve this problem, in 2009, Lee et al. [26] performed a simplification technique based on the tree leaf density of rendered images viewed from multiple angles in which the simplification process preserves different levels of densities on all partitions of a tree model.

### 2.2. Simplification Methods Based on Mutual Information

In many works, information entropy has been studied to measure the correlation between a set of viewpoints and the visibility of the objects, as can be shown in Viola et al. [27] and Feixas et al. [28]. They suggest that the variation on entropy has a close relationship with the model silhouette during the simplification process. Based on the measures proposed in viewpoint entropy by Vázquez et al. [29,30] and the viewpoint Kullback–Leibler distance introduced in [31], Castelló et al. [10] proposed a viewpoint-based simplification approach for polygonal meshes driven by an information-theoretic measure, viewpoint mutual information (VMI). This metric measures the correlation between a viewpoint and the set of polygons of the object or scene. The algorithm applies the best half-edge collapse as a decimation criterion and uses the variation in mutual information to measure the collapse error as a new simplification error metric. VMI decreases the visual error more for simplified meshes than viewpoint entropy, since it is able to maintain the silhouette better, and achieves the best results in visual similarity because it is able to remove all the invisible inner regions. VMI has been used to compute the best camera positions in volume rendering and polygonal meshes.

The works previously analyzed are related to the simplification of general meshes. However, a viewpoint-driven foliage simplification algorithm based on an information theoretic measure was proposed by Zhang [32] and Zhu et al. [33] that deals with the foliage of the plants. They reshape complex leaves as quadrilaterals. Then, the leaves are separated into clouds of cells for rapidly finding the best leaf pair to simplify. Two information-theoretic measures are evaluated in the simplification algorithm: mutual information and leaf visibility from a set of viewpoints. The simplification process consists of leaf-collapse operations, in which two leaves disappear and a new one appears based on the area maximum.

## 3. Viewpoint-Based Error Metric Using MI

The metric used to simplify the foliage of the plants in the presented foliage simplification method is based on the information-theoretic measure called viewpoint mutual information (VMI). The authors use mutual information (MI) in [34] as a shape descriptor for object recognition, which is suitable for capturing the shape variation. Moreover, works presented in [27,28] introduced VMI to select the best views, also demonstrating that the VMI depends on the shape of the object and not on the resolution of the mesh. Let *V* be a set of viewpoints and *O* the set of polygons of an object. Viewpoints will be indexed by *v* and polygons by *o*. Viewpoint entropy, based on Shannon entropy, has been defined [29] from the relative area of the polygons projected over the sphere of directions centered at viewpoint *v*.

The marginal probability distribution of *V* is given by $p\left(v\right)=1/N_{v}$, where $N_{v}$ is the number of viewpoints. That is, the same probability is assigned to each viewpoint, although other distributions could be used. Let $a_{o}$ be the area of the polygon *o* projected over the sphere, $N_{O}$ be the number of polygons of the object and $a_{t}=\sum_{i=0}^{N_{O}}a_{i}$ the total area of the projected polygons over the sphere. The conditional probability $p\left(o|v\right)=a_{o}/a_{t}$ is defined by the normalized projected area of polygon *o* over the sphere of directions centered at viewpoint *v*. Finally, the marginal probability distribution of *O* is given by $p\left(o\right)=\sum_{v\in V}p\left(v\right)p\left(o|v\right)=\left(1/N_{v}\right)\sum_{v\in V}p\left(o|v\right)$. Finally, VMI is defined according to Equation (1), which represents the degree of correlation between the viewpoint *v* and the set of polygons *O*.

(1) $$I\left(v;O\right)=\sum_{o\in 0}p\left(o|v\right)\log\frac{p(o|v)}{p(o)}$$

According to [27,28], the difference shown in Equation (2) quantifies for a given viewpoint *v* the variation in the shape of that polygonal object *O* to $O^{\prime }$, viewed from that point of view.

(2) $$\left|I\left(v;O\right)-I\left(v;O^{\prime }\right)\right|$$

The work presented by [10] evaluates every simplification step that changes a polygonal object *O* to $O^{\prime }$ performing edge collapse in the geometry. The error associated with every one of these collapse operations $C_{e}$ is defined by the sum of variations in VMI for all viewpoints (Equation (3)).

(3) $$C_{e}=\sum_{v\in V}\left|I\left(v;O\right)-I\left(v;O^{\prime }\right)\right|$$

In this work, this idea is applied to measure the variation of the shape of the foliage *F*, not composed by continuous meshes. As in [10], a set of viewpoints *V* has been considered in order to cover the object from different views. The distribution of the cameras around the foliage has been performed taking into account that VMI is sensitive to the distance of the object. In our case, the considered object is the foliage of a plant, so a simplification operation involves the elimination or removal of a leaf. Taking these facts into account, the error introduced by a leaf removal $L_{pru}$ is defined by the sum of the VMI variations for all the viewpoints *v* in *V*:

(4) $$L_{pru}\left(l\right)=\sum_{v\in V}\left|I\left(v;F\right)-I\left(v;F^{\prime }\right)\right|$$

where $F^{\prime }$ represents the foliage without the visualization of the leaf *l*.

Moreover, following the method employed in [10], the technique for computing viewpoint entropy based on projected areas is the hybrid SW-HW histogram. This technique takes advantage of the PCI Express bus symmetry. A different color is assigned to each leaf, composed by a combination of polygons where the image of the leaf is textured, and the whole object is sent for rendering. Next, a buffer read operation is performed, and then, this buffer is analyzed pixel-by-pixel to retrieve data about its color. Using RGBa color encoding with a byte value for each channel, up to $256^{4}$ polygons can be calculated with only one single rendering pass. We used this technique during the simplification process.

Another concept to consider is the resolution of the images generated by every camera that have to be compared. The method implemented for estimating the error is based on the projected areas of polygons. Depending on the orientation of the leaves in the foliage, problems can appear when this error is obtained, especially in the case of tiny or long and thin triangles whose projections do not cover a single pixel. One possible solution to reduce these errors is to increase the image resolution, but this obviously penalizes the performance of our simplification method. According to the work [10], the optimum resolution of the images used to determine the error is 256×256. The authors in this work demonstrate that this resolution is the best option to balance time computing and shape preserving.

## 4. Simplification Scheme

**Figure 1:**
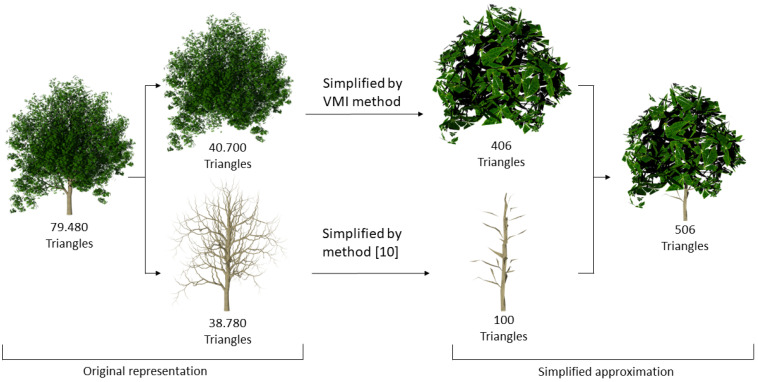
Workflow for obtaining a simplified approximation of a tree, finally formed by 506 polygons. VMI, viewpoint mutual information.

Trees and plants can be easily divided in two different parts: the solid component, i.e., the trunk and the branches, and the sparse component, the foliage or leaves. In order to offer a whole simplification of the plant, every part is separately analyzed and simplified. Figure 1 illustrates the process followed to achieve a coarser LoD.

The trunk is formed by a set of polygonal meshes. Work presented in [10] performs the viewpoint-based simplification of general meshes driven by the VMI, so this method has been applied to this part of the plant. Branch simplification, as is shown in the bottom of Figure 1, illustrates a result obtained applying this method.

**Figure 2:**
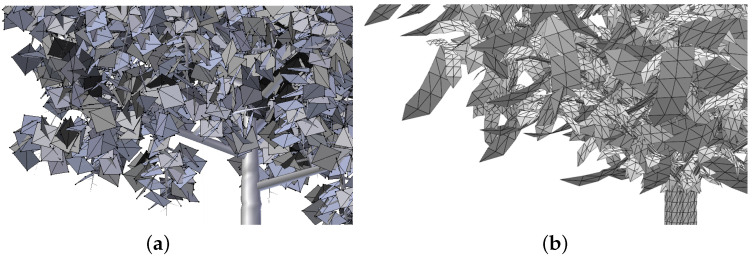
Example of leaves represented by different polygon combinations. (**a**) Leaf representation by quads; (**b**) leaf representation by the combination of polygons.

Foliage representation is different depending on the tool that has been used to generate the plant. Xfrog [35] and [36], two of the most popular software programs that deal with plant modeling, usually represent the leaves using a quad formed by two triangles or a combination of them. Some of these polygon combinations can be appreciated in Figure 2. This is a very common representation in trees that have been designed for interactive applications, such as video games.

In order to obtain an LoD of the foliage composed by a lower number of leaves, the simplification operation has to be performed iteratively until the desired number of leaves is achieved. In the case of the presented method, the simplification operation is the leaf removal.

### 4.1. Leaf Removal Operation

The simplification process is based on the leaf removal operation. As well as the work presented in [9], this operation implies eliminating a whole leaf of the foliage. In every iteration, our simplification method evaluates the associated cost to prune every one of the leaves in the foliage, comparing the obtained images with the images of the original plant and associating a cost to every pruning $L_{pru}$ (Equation (4)). The leaf firstly removed of the representation will be the one that introduces the lowest deviation in the canopy.

**Figure 3:**
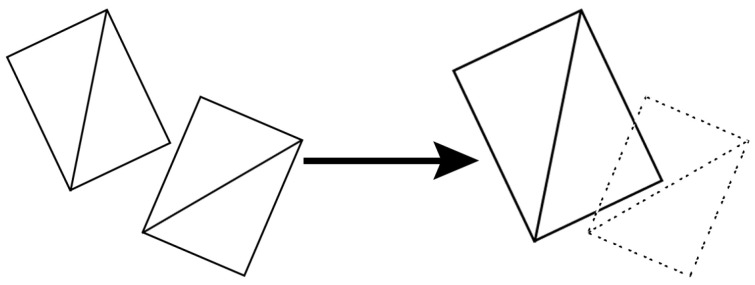
Example of resizing a leaf that covers the pruned one (drawn with dashes on the right).

Once the leaf that introduces the lowest cost is determined, the next step is to maintain the leafy appearance of the foliage. Then, its nearest leaf is located and its size scaled in order to cover an area that avoids appreciating the gap produced by the pruning in the final approximation. This nearest leaf is the one that makes the geometric distance between the center of the combinations of polygons that represent the leaves lowest. The developed method takes into account the size of the leaf to prune and the deviation $L_{pru}$ that is going to introduce to the final representation after its removal. Let *l* be the leaf to be pruned and $l^{\prime }$ its nearest leaf. Let Size(l) be a function that calculates the size of the leaf that it takes as input. Then, the size of the nearest leaf is altered following the equation shown in Equation (5). Figure 3 illustrates this process in order to for better comprehension.

(5) $$Size\left(l^{\prime }\right)=Size\left(l^{\prime }\right)+Size\left(l\right)*L_{pru}\left(l\right),\;L_{pru}\left(l\right)\in \left[0,1\right]$$

Leaves that introduce a very low error in the representation after their removal hardly vary relative to the size of the leaves nearest to them. By contrast, leaves that produce a high error after the disappearance of the visualization, compensate for this change of appearance by increasing the size of the nearest leaves in order to cover the opening that they have produced. In this case, the nearest leaf is resized to cover its own area and that of the pruned leaf.

### 4.2. Evaluating the Number of Viewpoints

The number of viewpoints condition the good quality of the results. However, it has to be considered that the more cameras are used, the higher the computing time to extract the LoD is. Works related to view-dependent simplification [10,21] demonstrate that in order to get a trade-off between accuracy and cost, it is appropriate to perform the measurements with 20 regularly-distributed viewpoints and rendered 256×256 resolution images. In this work, a study has been performed to analyze the number of cameras that allows us to obtain a better simplification. The foliage used in the test is shown in Figure 1, composed of 20,376 leaves (40,700 triangles).

**Figure 4:**
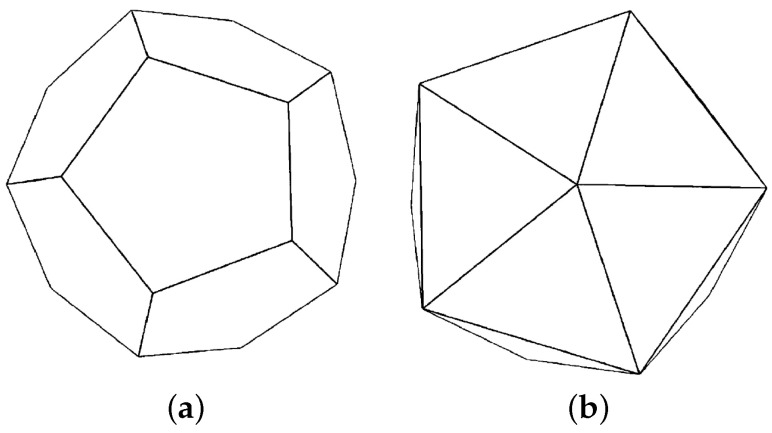
Camera positions that have been used in the test. (**a**) Dodecahedron: 20 vertices; (**b**) icosahedron: 12 vertices.

Different configurations have been tested in the experiment. First of all, 20 cameras were distributed on the vertices of a dodecahedron of one unit of radius that surrounds the object (shown in Figure 4a). Next, 12 view-points have been situated around the foliage following the vertices of a icosahedron (Figure 4b), and finally, a hybrid configuration of the cameras has also been tested, which we have named variable number of cameras (VNC). This last configuration takes into account the percentage of simplification that is going to be performed, establishing four ranges in the method. Simplifications in the range of [0%, 10%], 20 cameras, [11%, 30%] of simplification, 12 view-points, the range between [31%, 50%] 8 cameras, and finally, until 90% (6) cameras are located around the object.

To measure the quality of the simplified models, another 12 different viewpoints have been distributed around every representation. These cameras are distributed following the points of the icosahedron shown in Figure 4b. The VMI error was calculated using perspective projection, a 60 degrees field of view, a 3.0 radius for the viewpoint sphere and a 512×512 image resolution with flat shading. This fact makes the evaluation of the introduced error more exact.

**Figure 5:**
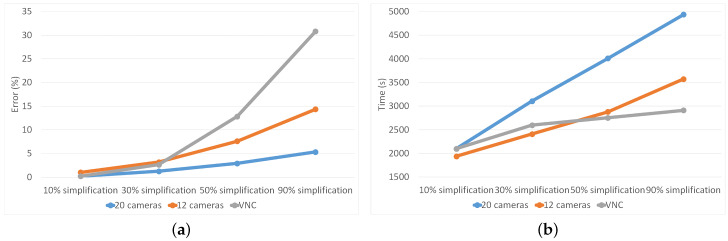
Camera positions that have been used in the test. (**a**) Error (percentage); (**b**) Time (measured in seconds). VNC = variable number of cameras.

Results are shown in Figure 5.

After testing the presented simplification method, the configuration of the 20 cameras has been found to be fairly satisfactory. The simplification process is an off-line process that is not performed in real time. The most wanted requirement in this kind of process is the quality of the results, regardless of the time required to get them. Figure 5a shows how the error of the resulting LoDs obtained by evaluating every leaf removal with 20 cameras is more similar to the original representation than that obtained with a lower number of cameras. Even when the obtained LoD has 90% less leaves than the original foliage, the error between both representations is about 5%. This process took 5000 s to complete, but the quality of the results are worth it.

### 4.3. Simplification Algorithm

The data structure that has been designed to perform the simplification of the foliage is shown next. The main one is the Foliage, which is compounded by a list of leaves. The data of every leaf are stored in the structure Leaf, where the edges that compound the leaf are referenced. Furthermore, the central point of the area that they occupy is stored in central_point, and the size of the leaf is stored in area_size. A list of all the edges of the foliage is stored in the structure Edge, where each one of the edges keeps the vertices that determine them. Finally, the data structure Vertices stores the geometric and the mapping coordinates.


		**Struct** Edge {
		Vertices[2] vertices;
		}
		**Struct** Vertices {
		float[3] vertex;
		float[2] uv;
		}
		**Struct** Leaf {
		Edge *edges;
		float[3] central_point;
		float area_size;
		}
		**Struct** Foliage {
		Leaf *leaves;
		}
		

The pseudocode of the implementation is shown in Algorithm 1. Due to the nature of the mesh that forms the foliage, the leaf removal cost has to be calculated every time a leaf removal is proposed. A removed leaf can modify the visibility of some leaves situated in different areas of the foliage, not only the nearest ones. Then, the VMI has to be computed every time a pruning is evaluated in order to obtain the cost of that simplification operation.

Let Leaves_To_Remain be the number of leaves that have to finally form the simplified representation of the foliage and number_of_leaves be a function that counts the number of leaves in the current representation. Every iteration of the algorithm will eliminate the leaf that introduces the lowest cost and will increase the size of the nearest one. Every time a leaf pruning is evaluated, the cost of this operation is checked in order to find the leaf that minimizes it. Then, this simulation is reversed to continue checking the rest of leaves in the representation. When all the leaves have been processed, the one with this minimum cost is pruned and its nearest leaf scaled. This process is repeated until the number of leaves that has to form the simplified LoD (Leaves_To_Remain) is achieved.
**Algorithm 1:** Process of leaf simplification.**1** // Initialize the number of cameras**2** n=20**3** // Compute the initial VMI for the foliage F**4** Compute I(v,F) where v={1,…,n}**5** // Initialize the set of leaves to evaluate**6** $F^{\prime }=F$**7** // Repeat the process until the number of leaves is the established number**8** while $\left(number\_of\_leaves\left(F^{\prime }\right))>Leaves\_To\_Remain\right)$ do**9**  min_cost=1000.0 // Initialize the cost**10**  for (l∈F)**11**   remove *l* to obtain $F^{\prime }$**12**   Compute $I(v,F^{\prime })$ where v={1,…,n}**13**   $L_{pru}\left(l\right)=\left|I(v,F)-I(v,F^{\prime })\right|$**14**   if $min\_cost>L_{pru}\left(l\right)$ then**15**    leaf_to_prune = *l***16**    $min\_cost=L_{pru}\left(l\right)$**17**   end if**18**   Undo removal**19**  end for**20**  // Remove leaf_to_prune**21**  $F^{\prime }=F^{\prime }-\left(leaf\_to\_prune\right)$**22**  // Obtain the nearest_leaf of leaf_to_prune**23**  $nearest\_leaf=Near\_Leaf(F^{\prime },leaf\_to\_prune)$**24**  // Scale the size of nearest_leaf according to Equation (5)**25**  nearest_leaf.area_size=Resize(nearest_leaf,l,min_cost)**26** end while

### 4.4. Algorithm Complexity

The total temporal cost of our algorithm depends on the number of viewpoints *V*, the number of pixels (image resolution) *P* and the number of leaves *L* in the representation. Calculating VMI implies a cost of O(PV). The cost of obtaining a coarser representation depends on the final number of leaves that remain. The pruning operation only has a cost of O(PVL). Finally, if we consider the worst case, that is all leaves are removed, the total complexity is $O(PVL^{2})$.

## 5. Results

Some experiments have been carried out in order to test the presented simplification scheme. They have been performed on a computer with an Intel Core i7-6700k 4.00 GHz with 16 GB RAM and NVIDIA GeForce CTX 1080 graphics card.

All the geometric models of plants that have been used in the tests have been modeled using the commercial modeling tool Xfrog [35]. Our method has been implemented taking advantage of the graphics hardware, so we have used for geometric visualization standard OpenGL running on current GPUs. To render our images, vertex buffer objects have been used, and for the off-screen rendering, the OpenGL frame buffer object extension has been employed.

### 5.1. Comparing the Simplified Approximations

**Figure 6:**
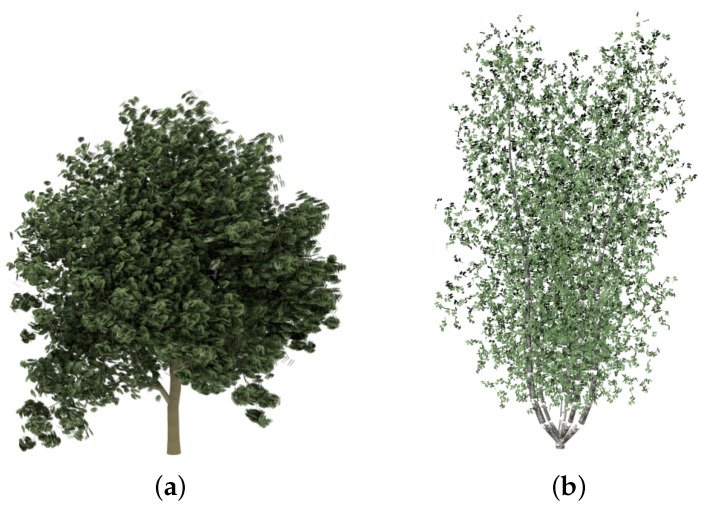
Trees and bushes used in the experiments that compare both methods. (**a**) English oak: 20,376 leaves; (**b**) *Carya illinoinensis*: 8140 leaves.

In order to evaluate the good quality of the results, some plants have been simplified and compared with the approximations obtained by applying the method presented by [6], called FSA (foliage simplification algorithm) by the authors, and an implementation of the stochastic method (SM) presented by Cook and Halstead [8]. The chosen models are shown in Figure 6.

**Figure 7:**
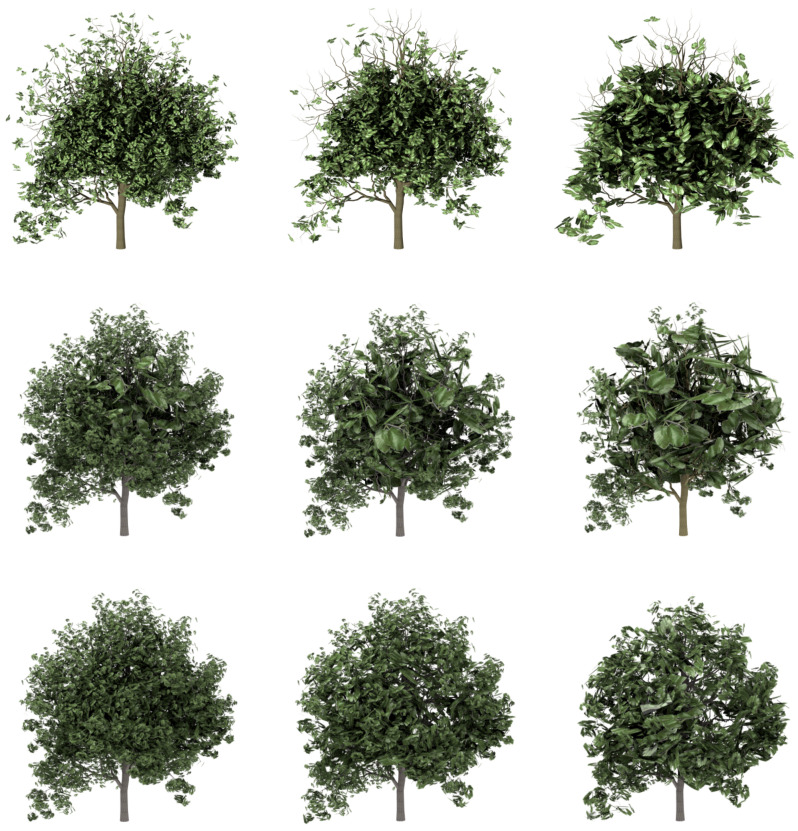
Visual comparison between the methods applied to the model tree shown in Figure 6a: **Top**. the stochastic simplification method (SM); **Middle**. FSA simplification; **bottom**. the VDS results. From left to right: 50%, 25% and 10%.

**Figure 8:**
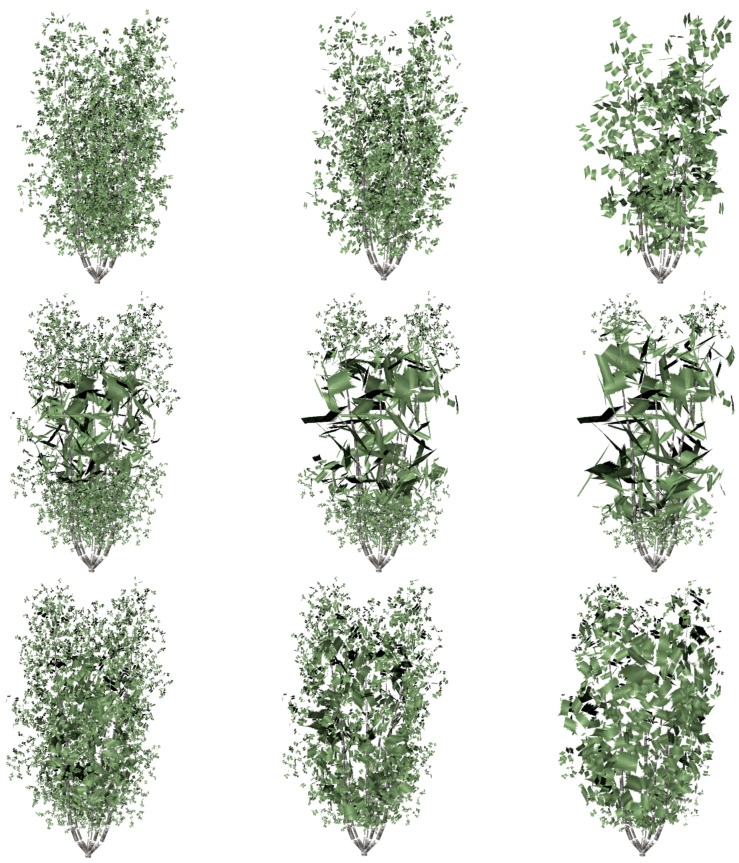
Visual comparison between the methods applied to the model tree shown in Figure 6b: **Top**. the SM; **Middle**. FSA simplification; **bottom**. the VDS results. From left to right: 50%, 25% and 10%.

Visual comparisons are shown in Figure 7 and Figure 8. Three levels of detail have been computed for every one of the plant models used in the test: the ones that approximately maintain 50%, 25% and 10% of the leaves. All the involved data are detailed in Table 1. The data related to the presented method are identified as VDS (viewpoint-driven simplification). First of all, the geometric details of every one of the plants that have been used in the test are described in it, adding the number of leaves of the resulting approximations.

Next, the quality of the simplified models has been measured using the mutual information (VMI). In this case, the final results obtained by both methods have been compared with the original models, taking the images from the 12 cameras situated around the approximations and original models. The percentage of introduced error is shown in the table for each one of the methods. It can be observed that the approximations obtained by the VDS method introduce a lower error than obtained by FSA and by SM. In fact, the approximations obtained by the SM method introduce the highest error because of its stochastic performance.

Finally, the computing time is also shown for every simplification. As can be seen, the cost of the presented method is quite higher than the time employed to obtain the levels of detail with the FSA and with the SM. View-dependent methods have in general a high temporal cost, but as was previously said, This is not really a problem because the calculations are performed in a pre-process [21]. This high cost is compensated by the good quality of the obtained results, as can be seen in the figures.

### 5.2. Evaluating the Simplified Approximations

**Figure 9:**
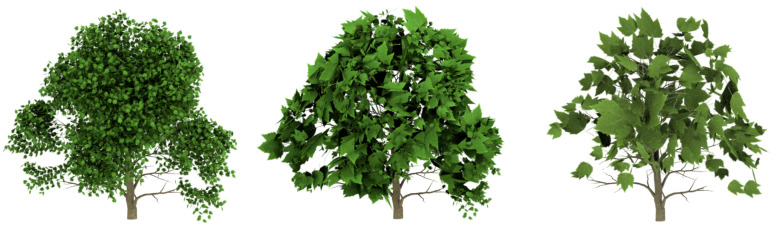
Visual results of the VMI simplification scheme for *Betula lenta*. From left to right: original model: 16,100 leaves; retaining 10% of the leaves: 1610; and retaining 1%: 161 leaves.

**Figure 10:**
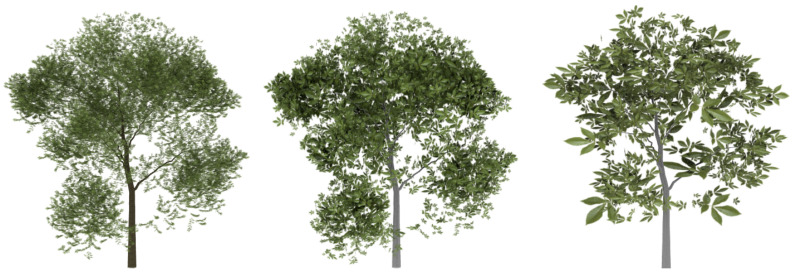
Visual results of the VDS scheme for *Alnus glutinosa*. From left to right: original model: 41,155 leaves; retaining 10% of leaves: 4115; and 1%: 411 leaves.

**Figure 11:**
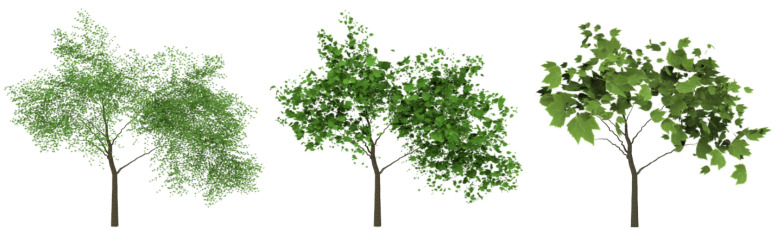
Visual results of the VDS scheme for *Betula populifolia*. From left to right: original model: 20,280 leaves; retaining 10% of leaves: 2028; and 1%: 202 leaves.

**Figure 12:**
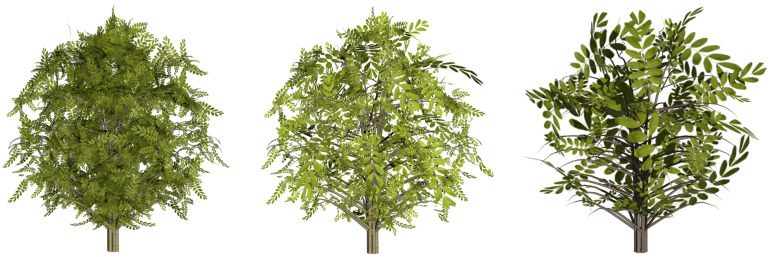
Visual results of the VDS scheme for *Sorbus aucuparia*. From left to right: original model: 2760 leaves (49,680 triangles); retaining 10% of leaves: 276; and 1%: 27 leaves.

**Figure 13:**
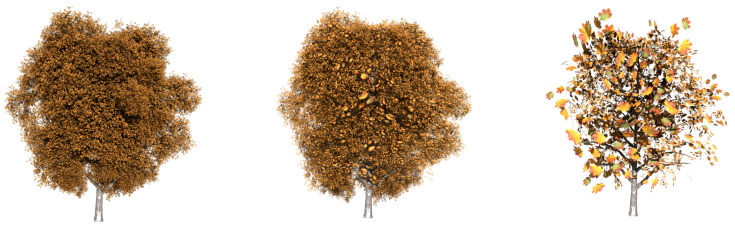
Visual results of the VDS scheme for the *Acer platanoides*. From left to right: original model: 105,688 leaves; retaining 10% of leaves: 10,569; and 1%: 1056 leaves.

Apart from the plant models that have been used to compare the VDS method, others have been simplified and the quality of the obtained results also evaluated using the same error measure, the VMI. Visual results are shown in Figure 9, Figure 10, Figure 11, Figure 12 and Figure 13. Three levels of detail have been obtained for every one of these plant models, the ones that approximately maintain 25%, 10% and 1% of the leaves. However, only the two last LoDs are included in the paper to evaluate the good visual quality. All the data are detailed in Table 2. The geometric details of every one of the plants that have been used in the test are described in it, adding the number of leaves of the resulting approximations. The leaves of the *Sorbus aucuparia* tree are composed by a combination of polygons, not only two triangles forming a quad (Figure 2b). Results obtained by simplifying this foliage model demonstrate the good performance of the method with models that use this kind of representation for the leaves. Finally, the percentage of VMI deviation against the original model is shown for every one of the computed levels of detail. Finally, the information of the computing time is also shown in this table for every simplification.

**Figure 14:**
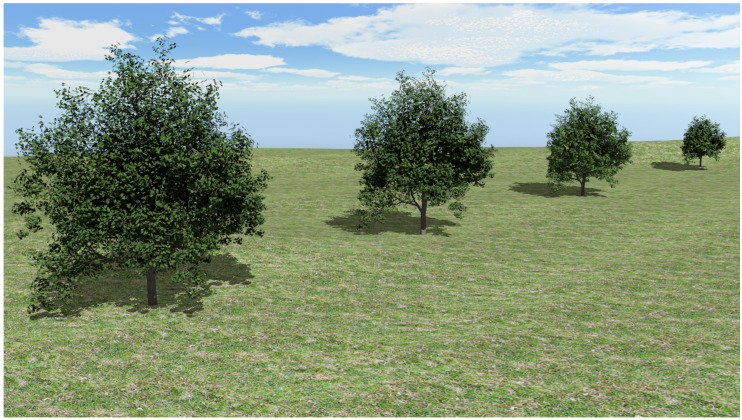
Example of the composition of a scene where different approximations of the same plant are shown (original foliage, 50%, 25% and 10% approximations).

Finally, a proposal of the composition of the different levels of detail shown in Figure 7 is illustrated in Figure 14. The distance to the camera has conditioned the choice of every approximation. As can be seen in the figure, the reduction of leaves in the representations cannot be appreciated even at the furthest level of detail.

### 5.3. Simplification Considering Visual Obstacles

**Figure 15:**
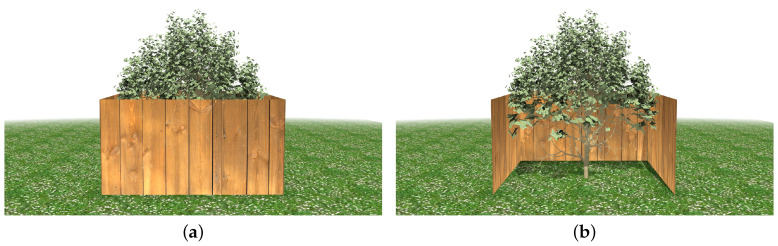
Simplified approximation of foliage when surrounded by a fence. (**a**) Level of detail formed by 10% of the leaves; (**b**) detail of the approximation making transparent the fence in front.

The last experiment that has been carried out takes some obstacles into account when the simplification is performed. In order to evaluate the good results in these cases, two different situations have been simulated. Firstly, the simplification method has taken as input the foliage of a tree that is partially hidden by a small fence that surrounds it (Figure 15). In this case, a set of leaves is completely hidden from some of the viewpoints. This makes the error introduced by pruning these leaves be negligible. If the remaining leaves have to be, for instance, 10% of the original representation, these non-visible leaves will be the first to be pruned, meaning that the leaves that remain are distributed in the discernible area of the canopy. Figure 15b shows the distribution of the leaves in a situation where these visual obstacles have been considered. For a better appreciation, the fence in front is rendered transparent.

**Figure 16:**
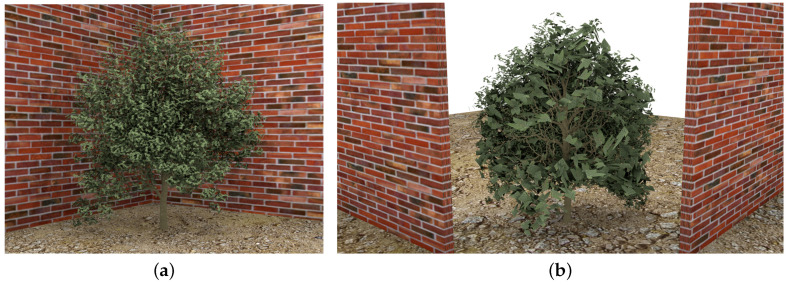
Simplified approximation of a plant when it is situated close to walls. (**a**) Ninety percent simplified; only 10% of leaves remain; (**b**) back view. Walls have been cut for a better appreciation.

Other kinds of visual obstacles that have also been tested are the walls situated near the plants. Some parts of the canopy are hidden by them, so leaves located in this area are the first pruned when the simplification is performed. Figure 16 illustrates this situation. As well as the previous example, only 10% of the leaves have been left at this level of detail. Figure 16b shows the back view of the approximation. The walls have been cut in order to appreciate that the leaves in the back area of the foliage have been pruned.

## 6. Conclusions and Future Work

This paper has presented a simplification method based on viewpoint mutual information that deals with the sparse part of the plants. The geometry that forms the canopy of the trees and plants is reduced by performing a viewpoint-driven simplification. In order to decrease the number of rendered leaves to a determined number, the visibility of every leaf in the canopy is evaluated according to viewpoint mutual information. To perform this evaluation, a set of cameras has been distributed around the foliage to evaluate the visual influence of each leaf pruning. Images obtained from these cameras are compared with the images of the original foliage, taken from the same viewpoints. This makes it possible to assign an error to every potential leaf pruning. The leaf that introduces the lowest error is pruned in every iteration.

The leafy appearance of the plant is preserved by resizing the nearest leaf of the removed one. The new size of this nearest leaf depends on the error introduced by the one that disappears. This resizing is addressed to fill the hole that has left the removed leaf, avoiding the pruning appearance. These processes are performed until the desired number of leaves is achieved. Different experiments have been carried out that demonstrate the good performance of the results, obtaining LoDs composed by a very low number of leaves that still maintain similarity with the original representation.

Combining the presented method with a general simplification scheme applied to the meshes that form the trunks and branches makes it possible to obtain different LoDs of plants and trees that can form a discrete multiresolution model. This set of approximations can be used in real time by video game designers in interactive applications. Real-time rendering can be achieved by changing the rendered representation from one LoD to another according to some criteria, such as the size of the foliage in the final rendering.

As future work, we are currently working on the construction of a continuous multiresolution model from this sequence of leaf removals. The fact that the presented method eliminates one leaf in every iteration makes it possible to store an ordered list of leaves taking the error that is introduced in the approximation into account. Storing this sequence in the GPU, it is easy to reduce or increase in a continuous way the number of leaves required by the visual application.

Another line of research that is being carried out is to obtain a simplification method that takes into account the skeletons to perform the simulation of the wind in the plants. By adding some restrictions to the presented method, such as associating each leaf with a skeleton or branch, this new scheme can be achieved. The presented method does not add any new leaves to the representation, so although some leaves are eliminated, the rest of the remaining leaves can perfectly maintain the link with the initial skeleton and simulate the movement of the wind.

## Figures and Tables

**Table 1:** Experimental data of the developed test comparing both simplification methods. VDS, viewpoint-driven simplification.

Model	Leaves		VMI Error			Time (s)		
	Original	Final	FSA	VDS	SM	FSA	VDS	SM
English Oak	20,376	10,175	12.01	3.71	19.75	98.32	4010.27	81.36
		5087	22.09	4.48	25.26	101.64	4501.14	83.64
		2035	45.17	5.31	32.25	105.93	4938.48	85.81
*Carya illinoinensis*	8140	4070	32.42	5.12	7.54	40.13	901.23	34.08
		2035	64.31	6.64	14.38	45.58	1018.97	37.18
		814	77.86	14.65	37.42	48.23	1231.29	39.92

**Table 2:** Experimental data evaluating the results of the presented method.

Model	Original		Final		VMI Error	Time (s)
	Triangles	Leaves	Triangles	Leaves		
*Betula lenta*	32,200	16,100	8050	4025	4.27	2412.80
			3220	1610	9.86	2634.06
			322	161	28.42	2782.18
*Alnus glutinosa*	82,310	41,155	20,576	10,288	10.53	9950.32
			8230	4115	14.54	10,301.56
			822	411	35.47	10,745.12
*Betula populifolia*	40,560	20,280	10,140	5070	12.29	3137.33
			4056	2028	19.84	3350.38
			404	202	39.14	3432.01
*Sorbus aucuparia*	49,680	2760	12,420	690	14.12	642.52
			4968	276	29.88	849.12
			486	27	51.40	912.41
*Acer platanoides*	211,376	105,688	52,844	26,422	9.75	14,112.47
			21,138	10,569	13.86	14,405.18
			2112	1056	37.15	14,484.03
